# Effect of Maternal Probiotic Intervention on HPA Axis, Immunity and Gut Microbiota in a Rat Model of Irritable Bowel Syndrome

**DOI:** 10.1371/journal.pone.0046051

**Published:** 2012-10-11

**Authors:** Javad Barouei, Mahta Moussavi, Deborah M. Hodgson

**Affiliations:** 1 Laboratory of Microbiology, School of Environmental and Life Sciences, the University of Newcastle, Callaghan, New South Wales, Australia; 2 Laboratories of Neuroimmunology, School of Psychology, the University of Newcastle, Callaghan, New South Wales, Australia; Charité, Campus Benjamin Franklin, Germany

## Abstract

**Objective:**

To examine whether maternal probiotic intervention influences the alterations in the brain-immune-gut axis induced by neonatal maternal separation (MS) and/or restraint stress in adulthood (AS) in Wistar rats.

**Design:**

Dams had free access to drinking water supplemented with *Bifidobacterium animalis* subsp *lactis* BB-12® (3×10^9^ CFU/mL) and *Propionibacterium jensenii* 702 (8.0×10^8^ CFU/mL) from 10 days before conception until postnatal day (PND) 22 (weaning day), or to control ad lib water. Offspring were subjected to MS from PND 2 to 14 or left undisturbed. From PND 83 to 85, animals underwent 30 min/day AS, or were left undisturbed as controls. On PND 24 and 86, blood samples were collected for corticosterone, ACTH and IgA measurement. Colonic contents were analysed for the composition of microflora and luminal IgA levels.

**Results:**

Exposure to MS significantly increased ACTH levels and neonatal fecal counts of aerobic and anaerobic bacteria, *E. coli*, enterococci and clostridia, but reduced plasma IgA levels compared with non-MS animals. Animals exposed to AS exhibited significantly increased ACTH and corticosterone levels, decreased aerobic bacteria and bifidobacteria, and increased *Bacteroides* and *E. coli* counts compared to non-AS animals. MS coupled with AS induced significantly decreased anaerobes and clostridia compared with the non-stress adult controls. Maternal probiotic intervention significantly increased neonatal corticosterone levels which persisted until at least week 12 in females only, and also resulted in elevated adult ACTH levels and altered neonatal microflora comparable to that of MS. However, it improved plasma IgA responses, increased enterococci and clostridia in MS adults, increased luminal IgA levels, and restored anaerobes, bifidobacteria and *E. coli* to normal in adults.

**Conclusion:**

Maternal probiotic intervention induced activation of neonatal stress pathways and an imbalance in gut microflora. Importantly however, it improved the immune environment of stressed animals and protected, in part, against stress-induced disturbances in adult gut microflora.

## Introduction

Early life stress has been reported to be associated with alterations to the development of the hypothalamic–pituitary–adrenal (HPA)-axis; a neuroendocrine system which is involved in the regulation of normal stress responses in vertebrates. Specifically early postnatal stress provokes dysregulations of HPA-axis function characterised by hyper-responsiveness to subsequent stress and long-term hyper-secretion of glucocorticoids [1]. This concept has been applied to the ontogeny of a variety of disorders later in life including functional gastro-intestinal disorders (FGIDs) such as irritable bowel syndrome (IBS) [2], [3]. Neonatal maternal separation (MS) in rodents is a well-established model of human brain-gut axis alterations induced by early life trauma. MS mimics cardinal features of human IBS [3], [4]. Previous research has shown that MS rats exhibit significantly higher corticosterone levels both in basal conditions and in response to a subsequent stress compared to the control animals [5], [6], [7], [8]. Furthermore, MS resulted in gut dysfunctions such as increased mucosal ion transport [6], [7], [9] and epithelial permeability to macromolecules [6], [7], [8], [9], [10], [11], [12], [13], increased mucosal adherent/penetrated commensal bacteria [6], [7] and bacterial translocation to internal organs [13], decreased intestinal mucin/mucus [12], [14], altered the integrity of gut microbiota [5], [12], [15], increased motility [9], [16] and increased visceral/colonic sensitivity [5], [11], [16], [17], [18], induced intestinal morphological/structural damage and increased mucosal mast cells and goblet cells [11], [13], [14].

MS was also associated with alterations in the immune system e.g. elevated levels of blood pro-inflammatory cytokines TNF-α and IFN-γ of MS rats exposed to subsequent open field stress in adulthood [5], increased activity of colonic myeloperoidase [11], [13] and increased colonic mRNA expression of cytokines IFN-γ, IL-1β, IL-2, IL-4, and IL-10 [13].

Neonatal probiotic intervention has been reported as a potential prophylaxis against the unfavourable imprinting induced on the brain-gut axis by MS in rats. It ameliorated MS-induced gut dysfunctions, restored gut flora and decreased elevated serum corticosterone levels [6].

Previous research has also shown that maternal introduction of probiotics not only results in colonisation of the neonatal gastrointestinal tract in animal models [19] and humans [20], [21], [22], but also causes substantial alterations in the offspring's gut microflora [20]. On the other hand, studies by Sudo and co-workers [23], [24] have shown that postnatal microbial colonisation programs the HPA stress response. Exaggerated HPA-axis stress responses to stress were observed in germ-free (GF) mice. Whilst the HPA-axis stress response was facilitated by intervention with *Escherichia coli*, this situation was reversed by administration with *Bifidobacterium infantis* [24]. It has also been reported that maternal administration of probiotics could modulate the immune system responsivity in a positive manner in offspring [25], [26].

Therefore, it could be expected that maternal probiotics intake could prevent or at least attenuate adverse outcomes of neonatal stress on intestinal barrier function in early and later life.

A combination of *Bifidobacterium animalis* subsp *lactis* BB-12® and *Propionibacterium jensenii* 702 was used in this study. *Bif. animalis* subsp *lactis* BB-12® was selected as it is the most widely recognised and extensively studied probiotic *Bifidobacterium* strain with a wide range of beneficial effects in human and animal models. In particular, this probiotic strain in combination with other probiotics has been reported to improve IBS symptom severity [27], decrease the composite IBS score (especially distension and abdominal pain), and stabilise gut microbiota in IBS patients [28]. *Bifidobacterium animalis* subsp *lactis* BB-12® was combined with *P. jensenii* 702 as the latter has been reported to increase populations of endogenous bifidobacteria in human subjects [29]. An *in vitro* study also demonstrated a mutual synergistic viability promoting effect of *P. jensenii* 702 and *Bif. animalis* subsp *lactis* BB-12® when co-cultivated [30]. This may enhance efficacy of the probiotic preparation.

The aim of the current study was to determine whether maternal probiotic intervention can protect against early and subsequent later life stress induced dysregulations of the brain-gut axis in Wistar rats. We demonstrated that exposure to MS or adult restraint stress (AS) resulted in alterations in HPA-axis activity. MS also suppressed plasma IgA responses and impaired normal balance of gut microflora. While maternal probiotic intervention appeared to activate neonatal stress pathways, it was associated with improved immune responses, and to some extent, protection of adult rats against abnormalities in the composition of gut microflora provoked by MS and/or AS exposure.

## Materials and Methods

### Animals and Animal Husbandry

Adult male and female Wistar rats were obtained from the University of Newcastle vivariums. Animals were housed in standard cages, 2–3 animals per cage lined with chip bedding on a 12∶12 hour light–dark cycle (lights on at 06:00 am) and an adjusted temperature at 23±1°C. Animals had free access to standard rat/mouse pellets (Specialty Feeds, Glen Forrest, WA, Australia) and drinking water. After acclimatisation for two weeks, two virgin females (12–20 weeks of age) were mated to a single male for 10 days. During the last week of gestation, dams were single housed. After delivery, dam and pups were housed together until weaning (PND 22). Offspring were then housed with same sex animals, 4–5 animals per box. All procedures were approved by the Animal Care and Ethics Committee of the University of Newcastle (ACEC No.: 1071). All efforts were made to minimise suffering.

### Study Design

Female breeders were randomly allocated to either ‘vehicle’ or ‘probiotic’ treatment groups. Dams in the probiotic treatment group had free access to drinking water supplemented with a probiotic preparation (details below) from 10 days before conception until and including weaning day. Control animals had equal access to water without the probiotics added. Newborns were subjected to neonatal maternal separation (MS) (details below) from PND 2 to 14 or left undisturbed. A subset of animals was euthanised at PND 24 with pentobarbiton sodium (Virbac Pty Ltd, Milperra, NSW, Australia) and blood and faecal samples were collected. The remaining animals were left undisturbed until PND 83 when they were exposed to three days restraint stress (30 min/day), and a 30 min isolation session at PND 86, or were left undisturbed as controls. All adult animals were then euthanised at PND 86 and blood and faecal samples were collected. Adult females were only tested in the diestrous phase to control for natural corticosterone variation within the estrous cycle [31]. Estrous cyclicity was monitored using a Rat Vaginal Impedance Checker (Muromachi Kikai, Tokyo, Osaka) according to the manufacturer's instructions.

### Probiotic Strains and Preparation

Freeze dried *Bifidobacterium animalis* subsp. *lactis* BB-12® was kindly provided by Chr. Hansen Pty. Ltd. Melbourne, Australia. *Propionibacterium jensenii* 702 isolated from raw bovine milk was obtained in lyophilised form the Laboratory of Food Microbiology, School of Environmental and Life Sciences, the University of Newcastle, Australia. The probiotic strains were recovered by two consecutive sub-cultures in appropriate media prior to use. *Bif. animalis* subsp. *lactis* BB-12 was grown overnight at 37°C in Reinforced Clostridial Medium (RCM) broth (Oxoid Australia Pty Ltd, Adelaide, Australia) under anaerobic conditions. *P. jensenii* 702 was grown anaerobically in yeast extract lactate (YEL) medium [32] at 30°C for 48 h. Bacterial cells were then harvested by centrifugation at 2000×*g* for 10 min and washed three times with Dulbecco's Phosphate-Buffered Saline (PBS, pH 7.0) (Gibco, Invitrogen Corp., Carlsbad, CA, USA). Bacterial pellets were then resuspended in PBS and refrigerated as probiotic stocks. Bacterial counts of stock cultures were determined by plating 100 µl aliquots of decimal dilutions of cultures on agar plates. *P. jensenii* 702 was counted on YEL agar after anaerobic incubation of the plates at 30°C for seven days. *Bif. animalis* subsp. *lactis* BB-12 was counted on TOS propionate agar (Yakult Pharmaceutical Ind., Co., Ltd, Tokyo, Japan) following anaerobic incubation of the plates at 37°C for two days. Fresh stock cultures were prepared every week. The probiotic drinking water was then prepared by the addition of both cultures to the drinking water so that the doses of *Bif. animalis* subsp. *lactis* BB-12 and *P. jensenii* 702 were approximately 3×10^9^ and 8.0×10^8^ CFU/mL respectively. The probiotic drinking water was available in 250-ml bottles for rats. The bottles were replaced with clean bottles containing fresh probiotic drinking water every day.

### Neonatal Maternal Separation (MS)

At PND 2, litters were randomly allocated into either ‘neonatal maternal separation’ (MS), or no stress condition. A slightly modified method of Barreau et al. [13] was used for MS stress. Pups in the MS condition were removed from litter mates and placed individually in 12×12×7 cm plastic containers lined with four layers of tissue paper and with holes in the sides allowing free air circulation. Containers were kept in a separate heated room (34±1°C) for three hours between 9:00 and 12:00 am from PND 2 to 14. Following the separation, pups were returned to their dams. Animals in the control groups were left undisturbed.

### Adult Stress Protocol (AS)

In adulthood animals were randomly allocated into either adult stress, or no stress condition. Animals allocated to the AS condition underwent a stress protocol which involved three consecutive days of restraint stress for 30 min/day (10:00–10:30 am, PND 83–85) using soft wire-mesh tubes with closed edges using binder clips, and one day of isolation housing (30 min, PND 86) as previously described [33]. During the period of AS, animals were food and water deprived. Animals' eyes were protected from light using thin light white paper towels. Rats in the ‘non-AS’ condition were left undisturbed.

### Corticosterone and ACTH Assays

Between 9:30 and 10:30am and prior to euthanisation, saphenous blood was collected into K_3_EDTA tubes (Greiner, Kremsmünster, Austria) and centrifuged at 1000×*g* for 20 min at 4°C. Supernatant (plasma) was then collected and stored at −20°C until assayed. Plasma corticosterone and ACTH levels were assessed respectively using a rat corticosterone ^125^I RIA kit (MP Biomedicals, Orangeburg, NY, USA) and a rat ACTH EIA kit (Phoenix Pharmaceuticals, Burlingame, CA, USA) according to manufacturer's instructions. Corticosterone and ACTH data were expressed as ng/mL and pg/mL respectively.

### Plasma and Luminal Immunoglobulin A (IgA)

Immediately following euthanisation, heart blood was collected via cardiac puncture. Plasma samples were then obtained by centrifugation and stored at −20°C until assayed. Frozen faecal pellets were thawed, weighed, and suspended in nine volumes (v/w) of a diluent supplied with Rat IgA ELISA kit (GenWay, San Diego, CA, USA). The mixture was incubated at 23°C for 15 min and vortex mixed. The suspension was then centrifuged at 12,000×*g* for 10 min, and supernatant was removed. This supernatant and plasma samples were diluted to final dilutions 1∶500–2000 and assayed for IgA levels using the Rat IgA ELISA kit (GenWay) according to the manufacturer's instructions. The optical densities were read at 450 nm using a Multiskan® EX microtiter plate reader (Thermo Electron Corp., Vantaa, Finland). Calculations were performed using Ascent software version 2.6 (Thermo). IgA data were expressed as µg/mL plasma or gr feces.

### Analysis of Faecal Aerobic and Anaerobic Bacteria

Fresh faecal pellets were collected directly from the rectum and distal colon of euthanised rats into 10 mL sterile tubes (Sarstedt, Nümbrecht, Germany) containing nine times (v/w) of an anaerobic dilution buffer [34] and homogenised by vortex mixing. Serial decimal dilutions were then prepared using the diluent. Amounts of 100 µl of diluted samples were plated on specific agar media. Total aerobes were enumerated on Tryptone Soya Agar (Oxoid, Basingstoke, Hampshire, UK) after a week of incubation at 37°C. Total anaerobes were counted on Glucose Blood Liver agar [35] after 48 h incubation at 37°C under anaerobic conditions using AnaeroGen W-Zip Compact (Oxoid). Bacterial counts were expressed as log_10_ CFU per gram of faecal samples.

### DNA Isolation and Purification

Fresh fecal pellets were also collected into sterile 1.8 mL cryogenic vials (NUNC, Roskilde, Denmark), snap frozen in liquid nitrogen and stored at −80°C. 100–200 mg of frozen samples was suspended in 1.4 ml buffer ASL (Qiagen, Hilden, Germany) in a microtube containing a 5 mm steel bead (Qiagen) and 300 mg acid washed 425–600 µm glass beads (sigma-Aldrich, Saint Louis, MO, USA). Samples were then completely homogenised using a TissueLyser LT (Qiagen) for 5 min at 50 Hz. DNA extraction/purification protocols were then followed as instructed in the QIAamp DNA Stool Mini Kit (Qiagen). To generate standard curves, DNA was also isolated from defined numbers of control bacterial cultures (serial decimal dilutions 10^0^–10^9^ CFU/mL) using the same kit. The concentration of DNA in final DNA eluate was measured using a NanoDrop 2000c (Thermo-Fisher Scientific, Wilmington, DE, USA). The DNA eluate was stored at −20°C until further analysis.

### Real-Time PCR Quantification of Faecal Microflora

Real-time PCR analysis of gut microflora was performed using a 7500 Fast real-time PCR System (Applied Biosystems, Foster City, CA). Previously reported primer sets and probes were used in this study (Table 1). Quantitative real-time PCR was performed using TaqMan and SYBR-Greens methods. Each reaction was carried out in a total volume of 25 µl containing 1× TaqMan Universal PCR Master Mix or 1× SYBR® Green PCR Master Mix (Applied Biosystems), 200 nM of both primers, 250 nM probe, 10 µL of diluted fecal DNA and 0.1 µg/µl bovine serum albumin (New England Biolabs, Ipswich, MA, USA). The amplification conditions were 10 min at 95°C (1 cycle), followed by 30 s at 95°C (40 cycles) and 1 min at 60°C (1 cycle). A melting step was added for SYBR-Greens amplifications. The obtained threshold cycles (Ct) were then used for calculation of fecal counts of bacterial targets using standard curves constructed by plotting defined counts of serially diluted control cultures versus their Ct.

**Table 1:** Primers and probes used for detecting bacterial groups in faecal samples.

Target	Primer sets and probes (5′–3′)	Method	Reference
*Bacteroides*	F: GAGAGGAAGGTCCCCCAC	TaqMan	[65]
	R: CGCTACTTGGCTGGTTCAG		
	P: CCATTGACCAATATTCCTCACTGCTGCCT		
Bifidobacteria	F: CGG GTG AGT AAT GCG TGA CC	TaqMan	[66]
	R: TGA TAG GAC GCG ACC CCA		
	P: CTC CTG GAA ACG GGT G		
Lactobacilli	F: GAGGCAGCAGTAGGGAATCTTC	TaqMan	[67]
	R: GGCCAGTTACTACCTCTATCCTTCTTC		
	P: ATGGAGCAACGCCGC		
Enterococci	F: CCCTTATTGTTAGTTGCCATCATT	SYBR-Green	[68]
	R: ACTCGTTGTACTTCCCATTGT		
*E. coli*	F: CATGCCGCGTGTATGAAGAA	SYBR-Green	[69]
	R:CGGGTAACGTCAATGAGCAAA		
*Clostridium* cluster XIVa	F: AAATGACGGTACCTGACTAA	TaqMan	[70]
	R: CTTTGAGTTTCATTCTTGCGAA		

F, Forward; R, Reverse; P, Probe.

### Statistical Analyses

Data analyses were performed using SAS® 9.2 (SAS Institute Inc., Cary, NC, USA). Generalised linear mixed model (GLMM) was used to analyse the design because of the hierarchical design and the mix of random and fixed variables. The design was a two-level hierarchical (pups nested within mothers) randomised design, unbalanced, and with incomplete blocks. The dam was a blocking variable which was included in the model as a random effect variable. Data sets (corticoaterone, ACTH, plasma and luminal IgA) were natural logarithm (Ln) transformed because data distributions of model residuals were not normal. A *p*-value≤0.05 was considered significant.

## Results

### HPA-axis activity

**Figure 1:**
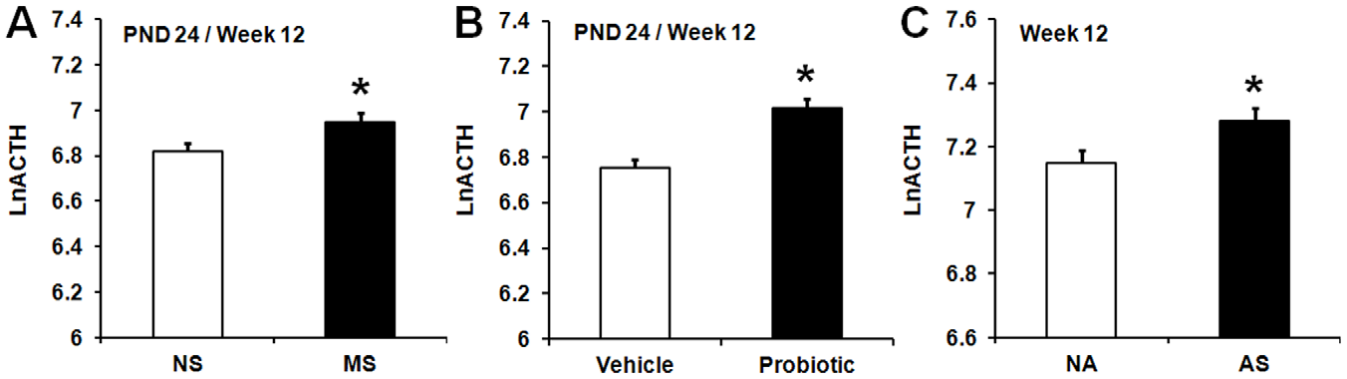
Effect of maternal probiotic intervention and stress on ACTH levels. **A**) Effect of neonatal maternal separation (MS) on natural log transformed plasma ACTH concentrations (LnACTH, least squares means+SE). Initial ACTH data were expressed as pg/mL plasma. The filled bar represents neonatally separated animals (MS, n = 107) and the hollow bar represents non-separated animals (NS, n = 111). **B**) Effect of maternal probiotic intervention on natural log transformed plasma ACTH concentrations (LnACTH, least squares means+SE). Hollow bar represents animals born to vehicle-treated dams (n = 111). Filled bar represents animals born to probiotic-treated dams (n = 107). **C**) Effect of adult stress on natural log transformed plasma ACTH concentrations (LnACTH, least squares means+SE). Hollow bar represents animals exposed to no adult stress (NA, n = 71). Filled bar represents animals exposed to adult stress (AS, (n = 72). An asterisk (*) indicates statistical significant difference (*p*≤0.05).

The model indicated significant main effects of MS [*F* (1,174) = 6.23, *p*<0.01], maternal probiotic intervention, [*F* (2,174) = 264.94, *p*<0.001] and AS [*F* (1,39) = 23.18, *p*<0.001] for ACTH. ACTH concentrations significantly increased in MS animals compared to non-MS animals (*p*≤0.05) (Figure 1A). Animals born to probiotic-treated dams also exhibited significantly greater ACTH concentrations compared with animals born to vehicle-treated dams (*p*≤0.05) (Figure 1B). In adulthood (week 12), animals subjected to AS exhibited significantly increased plasma ACTH levels compared with non-AS animals (*p*≤0.05) (Figure 1C).

**Figure 2:**
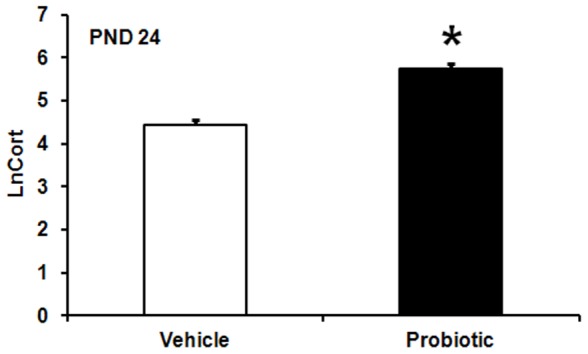
Effect of maternal probiotic intervention on neonatal corticosterone levels. Effect of maternal probiotic intervention on natural log transformed plasma corticosterone concentrations (LnCort, least squares means+SE) at PND 24. Initial corticosterone data were expressed as ng/mL plasma. The filled bar represents animals born to probiotic-treated dams (n = 40) and the hollow bar represents animals born to vehicle-treated dams (n = 45). An asterisk (*) indicates statistical significant difference (*p*≤0.05).

MS exposure did not affect corticosterone levels at PND 24 and in adulthood. The model indicated significant two-way interactions between AS, probiotic and sex [AS×probiotic, *F* (2,184) = 20.55, *p*<0.001; AS×Sex, *F* (2,184) = 6.27, *p*<0.002; probiotic×Sex, *F* (1,184) = 6.71, *p*<0.01]. At PND 24, pups born to probiotic-treated dams exhibited significantly higher corticosterone levels compared to pups born to vehicle-treated dams (*p*≤0.05) (Figure 2).

**Figure 3:**
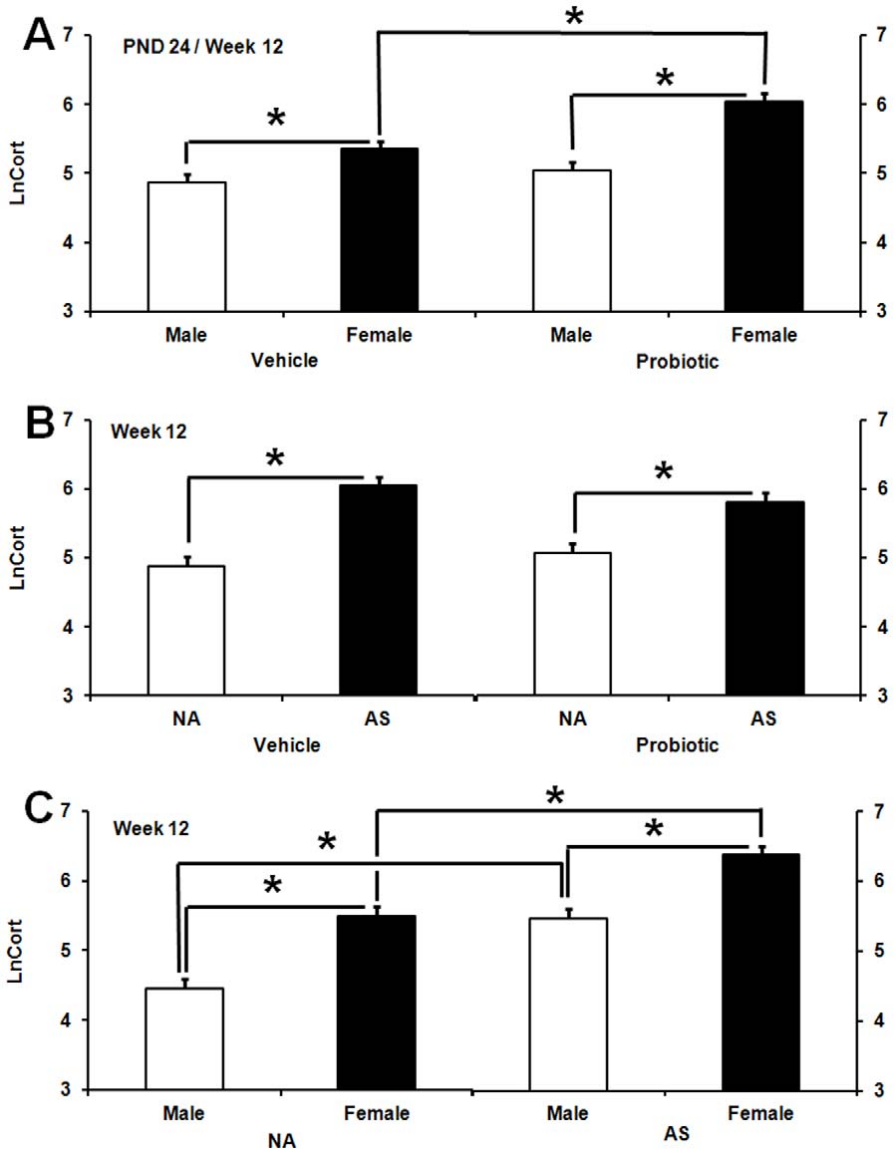
Effect of maternal probiotics, stress and gender on adult corticosterone levels. **A**) Effect of maternal probiotic intervention and sex on natural log (Ln) transformed plasma corticosterone levels (LnCort, least squares means+SE). Initial corticosterone data were expressed as ng/mL plasma. The figure presents aggregated data across test days (PND 24 and 86). Hollow bars represent males: male vehicle (n = 71), male probiotic (n = 53). Filled bars represent females: female vehicle (n = 62), female probiotic (n = 58). **B**) Effect of maternal probiotic intervention and adult restraint stress on Ln-transformed plasma corticosterone concentrations (LnCort, least squares means+SE) in adulthood (week 12).Hollow bars represent animals exposed to no-stress in adulthood (NA): NA vehicle (n = 39), NA probiotic (n = 35). Filled bars represent animals exposed to stress in adulthood (AS): AS vehicle (n = 39), AS probiotic (n = 36). **C**) Effect of adult restraint stress and sex on Ln-transformed plasma corticosterone concentrations (LnCort, least squares means+SE) in adulthood (week 12). Hollow bars represent males: NA male (n = 35), AS males (n = 36). Filled bars represent females: NA females (n = 39), AS females (n = 39). An asterisk (*) indicates statistical significant difference (*p*≤0.05).

Females born to probiotic- and vehicle-treated dams displayed significantly greater corticosterone levels compared to their male counterparts born to probiotic and non-probiotic treated dams (*p*≤0.05 in both cases) (Figure 3A). In addition, females born to probiotic-treated dams displayed significant higher levels of corticosterone compared with females born to vehicle-treated dams (*p*≤0.05).

AS and non-AS females also exhibited significantly higher levels of corticosterone compared with their respective males (*p*≤0.05 in both cases) (Figure 3C). Both males and females exposed to AS showed significant greater corticosterone levels compared to their respective non-AS animals (*p*≤0.05) (Figure 3C). AS exposure in animals born to either vehicle- or probiotic-treated dams significantly increased corticosterone concentrations relative to non-AS animals in vehicle and probiotic subsets (*p*≤0.05 in both cases) (Figure 3B).

### Plasma IgA responses

**Figure 4:**
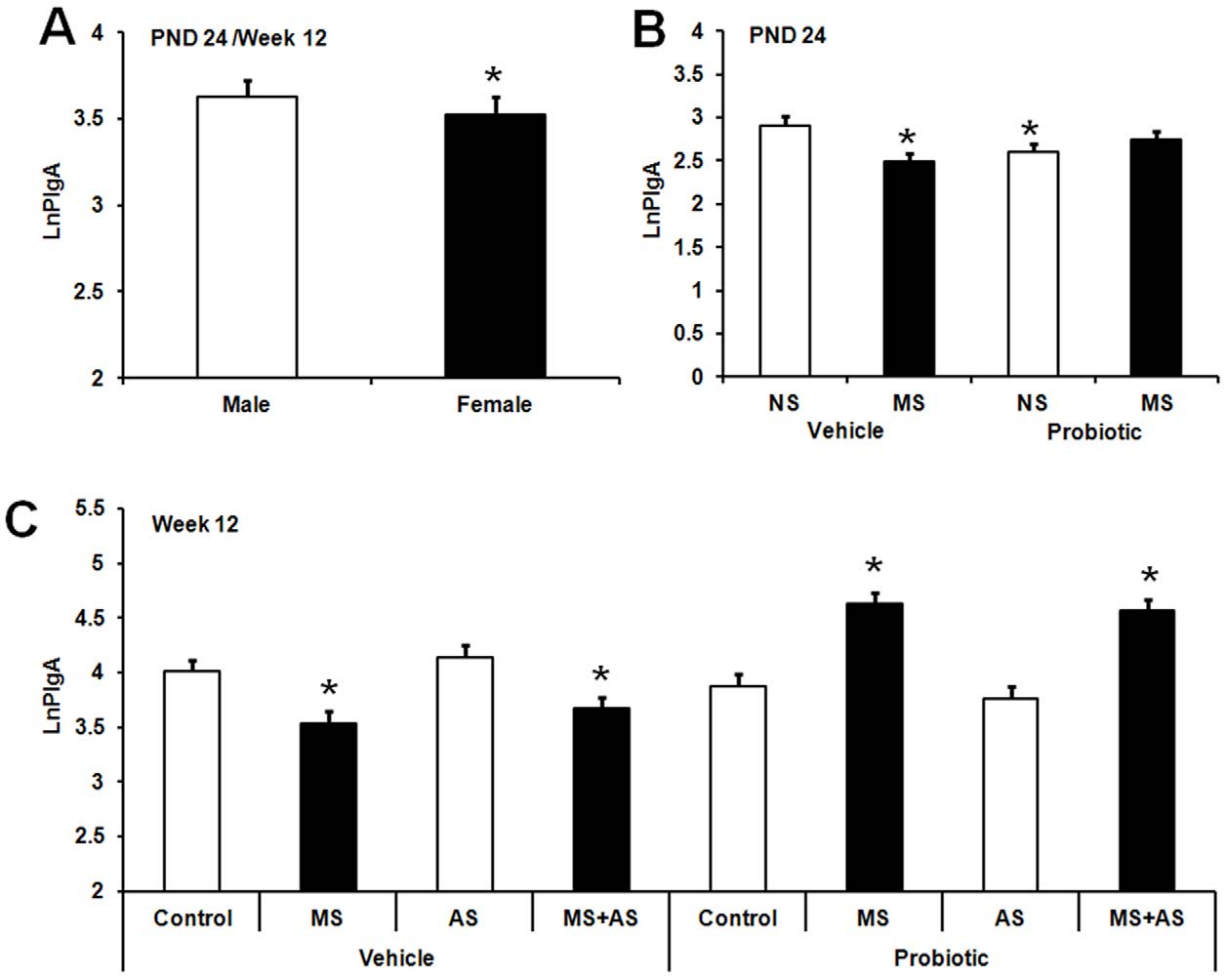
Effect of maternal probiotic intervention, stress, and gender on plasma IgA levels. **A**) Effect of sex on Ln-transformed plasma IgA concentrations (LnPIgA, least squares means+SE) in rats. The figure presents aggregated data across test days (PND 24 and 86). Initial IgA data were expressed as µg/mL plasma. The hollow bar represents males (n = 109) and the filled bar represents females (n = 117). An asterisk (*) indicates statistical significant difference (*p*≤0.05). **B**) Effect of maternal probiotic intake and neonatal maternal separation (MS) on Ln-transformed plasma IgA concentrations (LnPIgA, least squares means+SE) in rats at PND 24. The hollow bars represent animals exposed to no-MS (NS): NS vehicle (n = 15), NS probiotic (n = 19). The filled bars represent animals exposed to MS: MS vehicle (n = 19), MS probiotic (n = 20). An asterisk (*) indicates statistical significant difference relative to vehicle NS animals (*p*≤0.05). **C**) Effect of maternal probiotic intake, neonatal maternal separation (MS) and adult restraint stress (AS) on Ln-transformed plasma IgA concentrations (LnPIgA, least squares means+SE) in adult rats. Hollow bars represent no-stress control animals or those exposed to adult stress (AS): control vehicle (n = 21), AS vehicle (n = 20), control probiotic (n = 17), AS probiotic (n = 17). Filled bars represent MS or MS+AS animals: MS vehicle (n = 20), MS+AS vehicle (n = 20), MS probiotic (n = 18), MS+AS probiotic (n = 19). An asterisk (*) shows significant difference compared to control animals in the vehicle subset (*p*≤0.05).

A significant main effect of sex was observed with males exhibiting significantly greater plasma IgA (PIgA) levels compared to females, *F* (1,179) = 7.26, *p*<0.008 (Figure 4A). At PND 24, a significant MS×probiotic interaction was observed, *F* (3,179) = 3.6, *p*<0.015 (Figure 4B). MS pups born to vehicle-treated dams and non-MS pups born to probiotic-treated dams displayed significantly lower PIgA compared with non-MS pups born to vehicle-treated dams (*p*≤0.05). There was no significant difference between MS pups in the probiotic subset and non-MS pups born to vehicle-treated dams.

In adulthood, a significant three-way interaction between MS, maternal probiotic intervention and AS was observed, *F* (2,179) = 6.75, *p*<0.001 (Figure 4C). Planned comparisons revealed significantly reduced PIgA levels in MS animals either with or without AS exposure in the vehicle subset (*p*≤0.05). However these animals (MS and MS+AS) in the probiotic subset exhibited significant greater PIgA levels compared to control animals (*p*≤0.05) (Figure 4C).

### Fecal IgA

**Figure 5:**
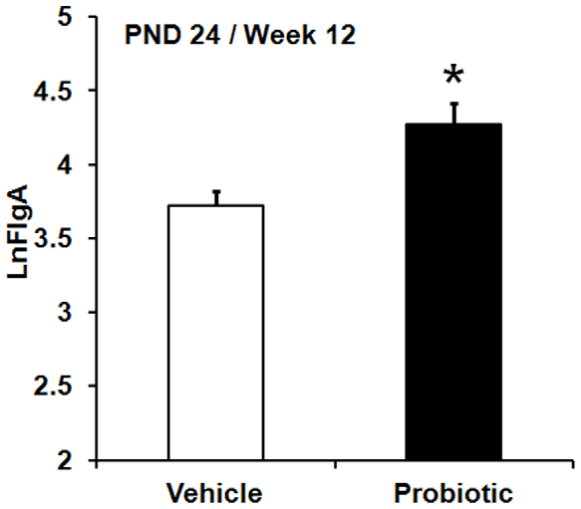
Maternal probiotic intervention and luminal IgA levels. Effect of maternal probiotic intervention on Ln-transformed faecal IgA concentrations (LnFIgA, least squares means+SE). Initial IgA data were expressed as µg/gr faeces. The figure presents aggregated data across test days (PND 24 and 86).The filled bar represents animals born to probiotic-treated dams (n = 101) and the hollow bar represents animals born to vehicle-treated dams (n = 113). An asterisk (*) indicates statistical significant difference (*p*≤0.05).

Neither neonatal stress, adult stress or a combination of both stresses affected faecal IgA concentrations, whereas a significant main effect of maternal probiotic intervention was observed for fecal IgA concentrations. This was significantly greater for animals born to probiotic-treated dams compared to animals born to vehicle-treated dams, *F* (1,38) = 10.56, *p*<0.002 (Figure 5).

### Gut microflora

**Figure 6:**
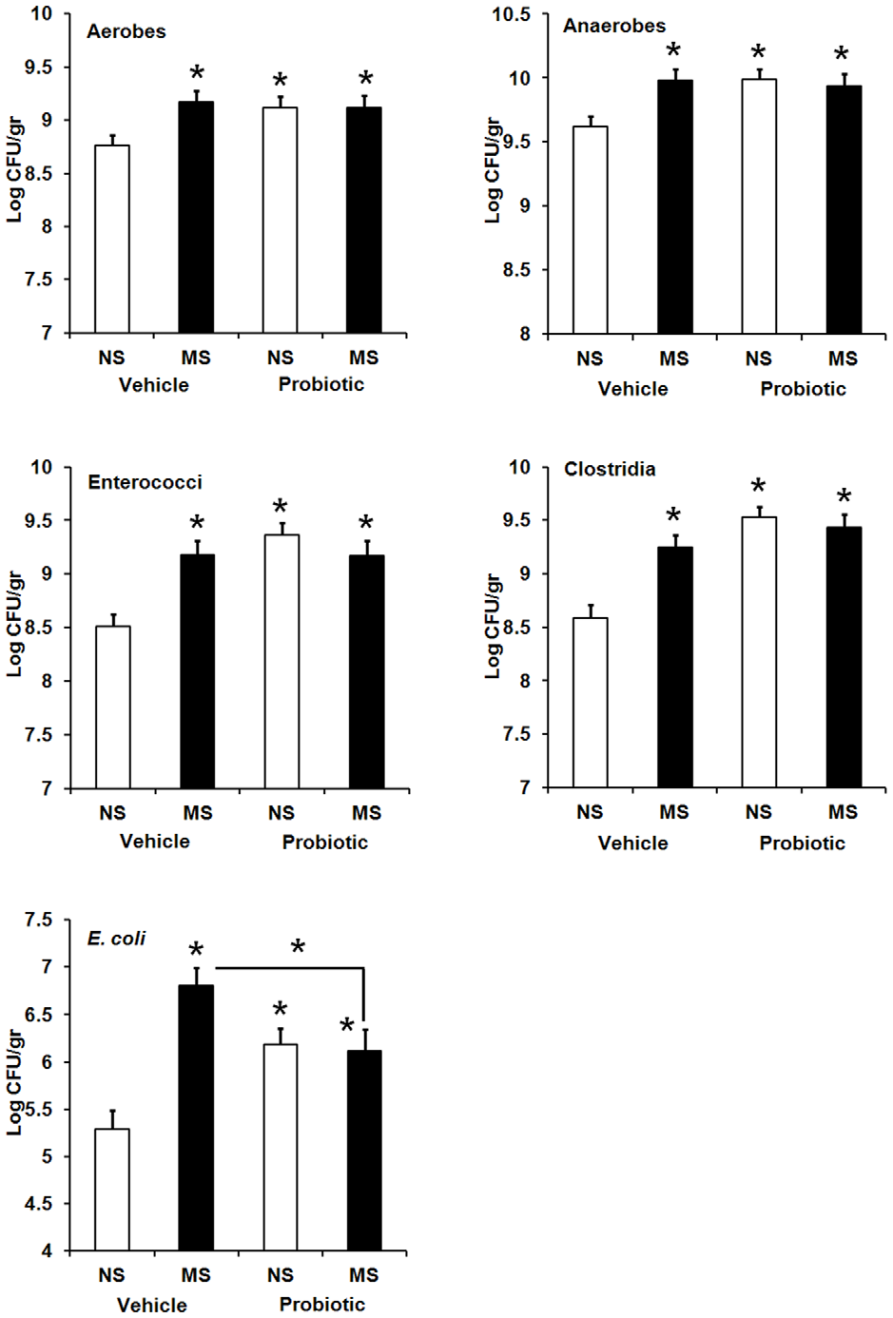
Effect of early life stress and maternal probiotic intake on neonatal gut microflora. Effect of neonatal maternal separation (MS) and maternal probiotic intake on composition of gut microflora (Log CFU/gr, least squares means+SE) in Wistar rats at PND 24. Hollow bars represent animals exposed to no neonatal stress (NS): NS vehicle (n = 14–20), NS probiotic (n = 19–20). Filled bars represent animals exposed to neonatal maternal separation (MS): MS vehicle (n = 19–20), MS probiotic (n = 20). An asterisk (*) shows significant difference compared to NS animals in the vehicle subset (*p*≤0.05).

At PND 24, fecal counts of lactobacilli, bifidobacteria and *Bacteroides* appeared to remain largely unaffected (data not shown). A significant interaction between MS and maternal probiotic intake was observed for all other five bacterial targets examined: aerobes, *F* (3,179) = 3.45, *p*<0.018; anaerobes, *F* (3,181) = 4.95, *p*<0.002; enterococci, *F* (3,181) = 9.95, *p*<0.001; clostridia *F* (3,170) = 12.09, *p*<0.001 and *E. coli, F* (3,162) = 10.17, *p*<0.001. Fecal counts of these bacteria appeared to significantly increase in MS pups born to vehicle-treated dams and both non-MS and MS pups from probiotic-treated dams relative to control pups in the vehicle subset (*p*≤0.05 in all cases) (Figure 6). While there was no significant difference between the three treatment groups with increased bacterial counts for four former bacterial groups, a significant reduction was observed in *E. coli* counts of both non-MS and MS pups born to probiotic-treated dams compared with that of MS pups born to vehicle-treated dams (*p*≤0.05). In this case *E. coli* counts did not return to control levels.

**Figure 7:**
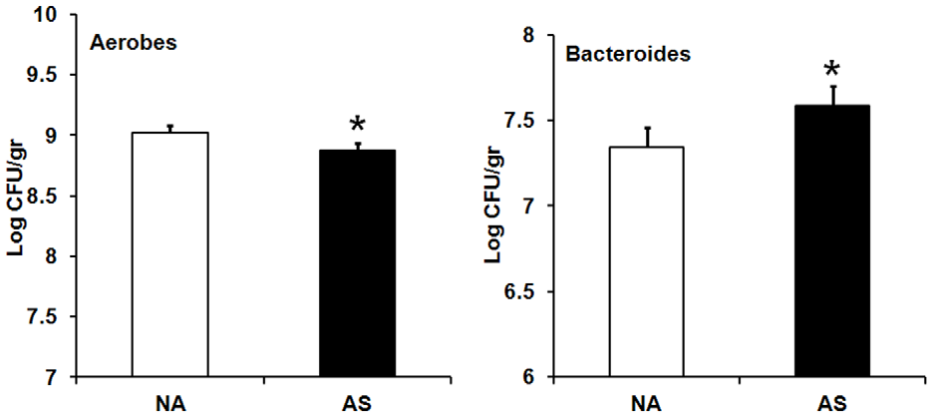
Effect of adult stress on faecal counts of aerobic bacteria and *Bacteroides*. Effect of exposure to stress in adulthood (week 12) on faecal counts of aerobic bacteria and Bacteroides (Log CFU/gr, least squares means+SE). The hollow bars represent animals exposed to no adult stress (NA): Aerobes NA (n = 72), *Bacteroides* NA (n = 67). The filled bars represent animals exposed to adult stress (AS): Aerobes AS (n = 75), *Bacteroides* AS (n = 75). An asterisk (*) indicates statistical significant difference (*p*≤0.05).

In adulthood (week 12), fecal counts of lactobacilli were not affected by maternal probiotic intervention, neonatal maternal separation, adult restraint stress or sex (data not shown). A significant main effect of AS was observed for counts of aerobic bacteria and *Bacteroides*. AS exposure resulted in significantly lower counts of aerobic bacteria [*F* (2,179) = 4.71, *p*<0.01], but higher counts of *Bacteroides* [(*F* (2,171) = 55.92, *p*<0.001)] compared with non-AS animals (Figure 7).

**Figure 8:**
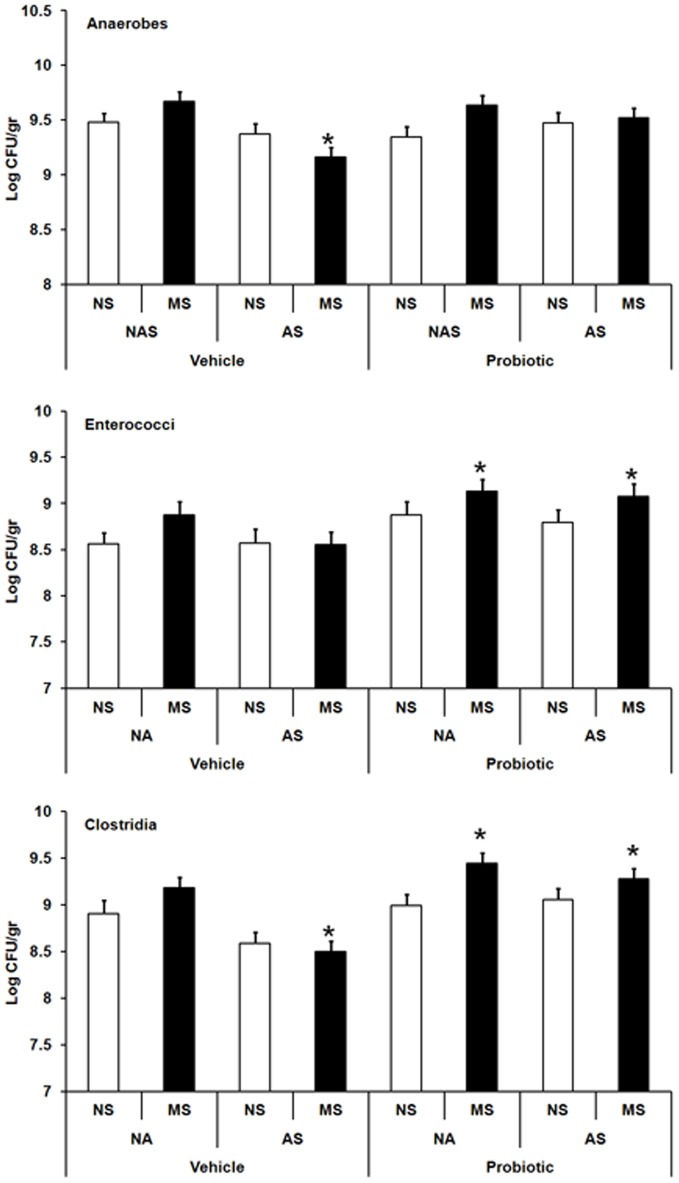
Effect of maternal probiotic intake and stress on adult gut microflora. Effect of maternal probiotic intake, neonatal maternal separation (MS) and adult restraint stress (AS) on faecal counts of anaerobes, enterococci and clostridia (Log CFU/gr, least squares means+SE) in Wistar rats at week 12. Hollow bars represent no-stress control animals or those exposed to adult stress (AS): control vehicle (n = 11–19), AS vehicle (n = 18–20), control probiotic (n = 17), AS probiotic (n = 17). Filled bars represent MS or MS+AS animals: MS vehicle (n = 20), MS+AS vehicle (n = 20), MS probiotic (n = 18), MS+AS probiotic (n = 19). An asterisk (*) shows significant difference compared to non-stressed (NS-NA) animals in the vehicle subset (*p*≤0.05).

Significant three-way interactions between maternal probiotic intake, MS and AS were observed for anaerobes, *F* (3,181) = 4.9, *p*<0.003; enterococci, *F* (7,181) = 4.27, *p*<0.001 and clostridia, *F* (2,170) = 5.22, *p*<0.001. MS animals exposed to AS displayed significantly lower anaerobes and clostridia counts compared with vehicle controls (*p*≤0.05 in both cases). Maternal probiotic intervention however, was associated with restoration of anaerobes in MS+AS animals and significant increases in enterococci and clostridia in MS and MS+AS animals compared with vehicle-control animals (*p*≤0.05 in all cases) (Figure 8).

**Figure 9:**
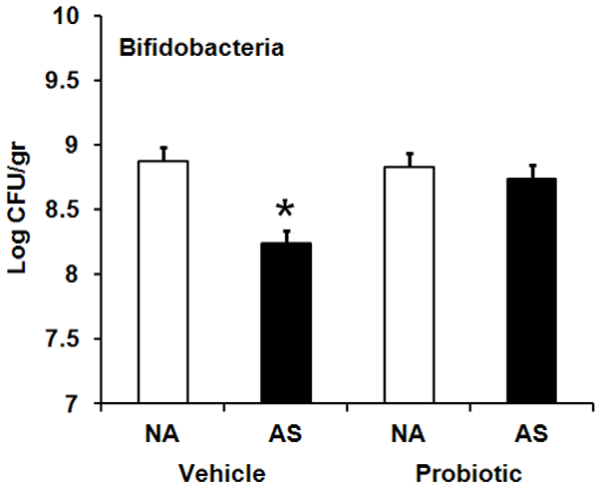
Effect of maternal probiotic intake and adult stress on bifidobacteria. Effect of maternal probiotic intake and adult restraint stress (AS) on faecal counts of bifidobacteria (Log CFU/gr, least squares means+SE) in Wistar rats at week 12. Hollow bars represent animals exposed to no adult stress (NA): NA vehicle (n = 33), NA probiotic (n = 35). Filled bars represent animals exposed to adult stress (AS): AS vehicle (n = 39), AS probiotic (n = 36). An asterisk (*) shows significant difference compared to non-stressed (NA) animals in the vehicle subset (*p*≤0.05).

In addition, a significant AS×probiotics interaction was observed for bifidobacteria, *F* (2,173) = 11.16, *p*<0.001. Bifidobacteria counts significantly declined in AS animals born to vehicle-treated dams compared with non-AS animals (*p*≤0.05), but were restored in the probiotic subset (Figure 9).

**Figure 10:**
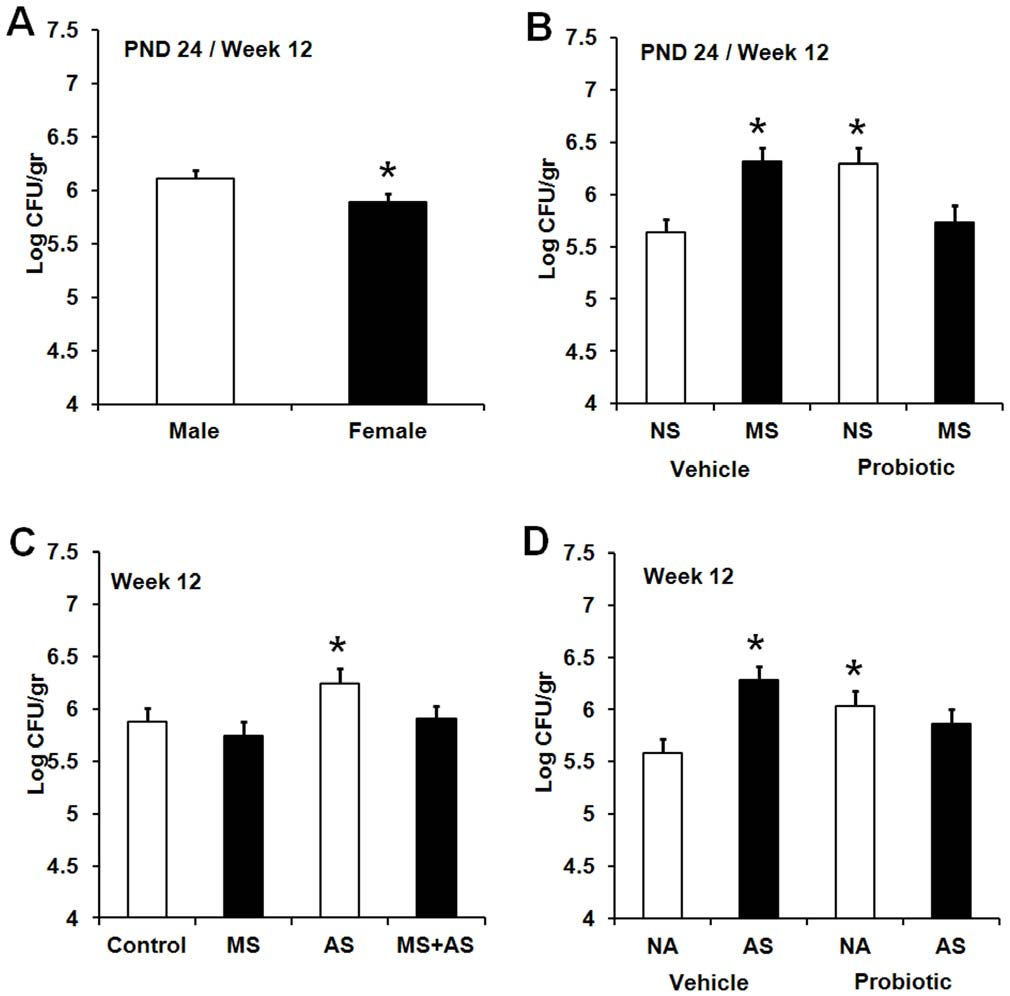
Effect of maternal probiotic intake, stress and gender on faecal counts of *E. coli*. **A**) Effect of sex on faecal counts of *E. coli* (Log CFU/gr, least squares means+SE) in rats. The figure presents aggregated data across test days (PND 24 and 86).The hollow bar represents males (M, n = 109) and the filled bar represents females (F, n = 112). An asterisk (*) indicates statistical significant difference (*p*≤0.05). **B**) Effect of maternal probiotic intake and neonatal maternal separation (MS) on faecal counts of *E. coli* (Log CFU/gr, least squares means+SE). The figure presents aggregated data across test days (PND 24 and 86).The hollow bars represent animals exposed to no-MS (NS): NS vehicle (n = 52), NS probiotic (n = 51). The filled bars represent animals exposed to MS: MS vehicle (n = 60), MS probiotic (n = 56). An asterisk (*) indicates statistical significant difference relative to vehicle NNS animals (p≤0.05). **C**) Effect of MS and AS on faecal counts of *E. coli* (Log CFU/gr, least squares means+SE) in adult Wistar rats (week 12). The hollow bars represent no-stress control animals or those exposed to AS: control (n = 33), AS (n = 37). The filled bars represent animals exposed to MS or MS+AS: MS (n = 38), MS+AS (n = 39). An asterisk (*) indicates statistical significant difference compared to the control (*p*≤0.05). **D**) Effect of maternal probiotic intake and AS on faecal counts of *E. coli* (Log CFU/gr, least squares means+SE) in adult Wistar rats (week 12). The hollow bars represent animals exposed to no-AS (NA): NA vehicle (n = 38), NA probiotic (n = 33). The filled bars represent animals exposed to AS: AS vehicle (n = 40), AS probiotic (n = 36). An asterisk (*) indicates statistical significant difference relative to vehicle NAS animals (*p*≤0.05).

In regard to *E.coli* counts, a significant main effect of sex was observed, *F* (1,174) = 8.86, *p*<0.003. Males displayed significantly higher counts of *E. coli* compared with females (*p*≤0.05) (Figure 10A). Furthermore, a significant MS×probiotics interaction across time was observed, *F* (1,174) = 21.44, *p*<0.001. *E.coli* counts significantly increased in vehicle MS animals compared with that of non-MS animals (*p*≤0.05). While bacterial counts returned to normal in the probiotic subset, probiotic non-MS animals exhibited higher number of *E.coli* compared with vehicle non-MS animals (*p*≤0.05) (Figure 10B).

In adulthood, significant two-way interactions between AS and MS or maternal probiotic intervention were observed, AS×MS, *F*(2,174) = 13.02, *p*<0.001; AS×probiotics, *F* (2,174) = 8.34, *p*<0.003. AS exposure significantly increased counts of *E. coli* compared to controls (*p*≤0.05) (Figure 10C). Maternal probiotic intervention was shown to return significantly increased *E. coli* counts in AS animals to that of non-AS animals born to vehicle-treated dams (Figure 10D). Maternal probiotic intervention however, was associated with significantly increased *E. coli* counts in non-AS animals relative to vehicle non-AS animals (*p*≤0.05)

## Discussion

This study aimed to examine the impact of maternal probiotic intervention on HPA-axis, immune and gut microflora alterations induced by early life and/or subsequent adult stress in Wistar rats. Previous research has shown that exposure of rats to neonatal maternal separation predisposes animals to brain-gut axis dysfunction in response to a subsequent adult stress [5], [9], [17], [36]. Essentially these stress models mimic to some extent that which is proposed to account for IBS i.e. stress in early life combined with stress in later life. This can be referred to as a Stress Diathesis model in which priming by early stress is compounded by stress in later life. In this study acute restraint stress in adulthood was used as a later-life stress due to its adverse effects on the brain-gut axis function in rodents [24], [37], [38], [39].

The data presented here provided evidence of adverse effects of neonatal maternal separation and/or adult restraint stress on HPA-axis, immune system and the balance of gut microflora. While maternal probiotic intake activated neonatal stress pathways which persisted into adulthood, it appeared to protect animals against immune dysfunction and to some extent against disturbance of the adult gut microflora provoked by neonatal maternal separation and/or adult restraint stress.

We demonstrated that neonatal maternal separation induced alterations in ACTH but not corticosterone responses. Maternal separation-induced ACTH elevations have previously been reported in the literature [40]. The lack of comparable changes in both corticosterone and ACTH is most likely due to the inability to optimise the timing of blood collection, due to experimental constraints, for both measures [33]. The reason for the lack of neonatal corticosterone response could also be due to a 10 day time lag between cessation of neonatal maternal separation and blood collection. A previous study reporting differences in corticosterone measured it one day after completion of exposure of Sprague-Dawley rats to neonatal maternal separation [6]. It has been also reported that the corticosterone level is maximal at day 20 (separation between PND 4–19) and the increase persists for 10 days. However corticosterone levels were lower than that of PND 20 [6]. These contrary findings are most likely due to differences in rat strains and neonatal separation protocols.

In support of previous research [24], [41], the present study found that exposure of animals to adult stress elevated both ACTH and corticosterone levels. Gender-dependent differences were also observed with females displaying higher basal and adult stress-induced corticosterone levels compared to their male counterparts. This is consistent with previous research [42], [43], [44].

Unexpectedly we found that maternal probiotic use is capable of inducing long-lasting hyperactivity of the HPA-axis as indicated by elevated neonatal corticosterone levels which persisted in female adults, and increased adult ACTH levels. While research has not yet investigated potential adverse effects of maternal introduction of probiotics on stress pathways, previous studies have shown such an increase in basal corticosterone levels of adult rats born to dams challenged with Gram negative bacterial lipopolysaccharides [45]. Thus, it is possible that probiotics are perceived as immune challenges which produce long-lasting alterations to the HPA-axis.

Stress has also been associated with immune system dysfunction because the immune system interfaces bidirectionally with the HPA-axis [46]. Mucosal and serum IgA play an important role as the first and second line of defence against antigens at mucosal surface and those which have breached the mucosal surface [47], [48], [49], [50], [51]. As such we investigated the effect of early and later life stress on serum and luminal IgA levels and whether maternal probiotic intervention can affect stress-induced IgA responses.

The present study found that neonatal maternal separation causes persistent reductions in plasma IgA levels. This is supported by previous research that early life adverse events such as early weaning in piglets resulted in low serum IgA levels [52]. It has been previously demonstrated that neonatal maternal separation in rats increases bacterial penetration into intestinal mucosa [6], [7], which is an initiating event for bacterial translocation to bloodstream causing blood and organ infection. Therefore increased bacterial penetration along with suppressed serum IgA production may potentially lead to an increased risk of infection in neonatally stressed animals. Interestingly the current study demonstrated that maternal probiotic intervention appeared to restore plasma IgA levels to normal in stressed neonates and reversed the decline in plasma IgA levels of neonatally stressed adults to well above control levels. While this has not been previously reported, it has been shown that probiotics enhance serum IgA responses in rodents exposed to bacterial pathogens [53], [54]. Therefore, maternal probiotics seem to contribute to increased immune defence capacity against penetrated/translocated bacteria in stressed animals through increased plasma IgA levels.

The data in the current study demonstrated that neonatal and adult stress did not affect luminal IgA levels; however maternal probiotic intervention induced significant elevations in fecal IgA concentrations. Previous research has shown that administration of probiotics to rodents increases intestinal mucosal IgA concentrations [55]. Mucosal IgA confines penetration of bacteria across the intestinal epithelial layer [56], [57], reduces mucosal-associated bacterial numbers and enhances the homeostasis of intestinal commensal microflora [58]. Therefore, our finding that maternal probiotics increased luminal IgA levels means that maternal probiotic administration enhances intestinal mucosal immune function in the maternally separated rat model of IBS where there is a high risk of increased intestinal permeability [6], [7].

The change in IgA does not appear to be linked to change in HPA axis activity, the observation which is inconsistent with previous studies [59], [60]. While the reason for this lack of linkage remains unknown, this is likely due to difference between blood collection optimum time points for detection of HPA axis and IgA responses.

Given that maternal probiotic intervention elevated luminal IgA known to be capable of promoting the homeostasis of intestinal commensal microflora, we also investigated whether this excessive IgA production could impact microbial alterations induced by stress.

Consistent with previous studies [5], [12], [15], we demonstrated that the normal balance of gut microflora is altered in maternally separated animals. Maternal separation caused shifts in neonatal gut microflora as indicated by fostering an overgrowth of total aerobes and anaerobes, and particularly potential negative bacteria *E. coli*, enterococci and clostridia, while beneficial bacteria such as lactobacilli and bifidobacteria remained unchanged. Interestingly maternal probiotic use induced comparable neonatal microflora changes to those seen in maternally separated pups born to vehicle-treated dams, an observation which has been previously reported but with probiotics administered neonatally [12]. Alterations to neonatal gut microflora could be explained by the fact that postnatal developing microbiota is extremely fragile and susceptible to environmental factors [61]. In addition, during this time, luminal IgA dynamically interacts with the development of gut microbiota and declines in some time points to allow bacterial colonisation [62]. Therefore events such as neonatal stress and maternal probiotics could potentially affect this process and lead to an imbalance in microflora.

While neonatally stressed adults exhibited no change in their gut microflora, exposure of these animals to an acute stressor in adulthood reduced faecal counts of anaerobic bacteria and clostridia. This means that early life stress sensitises specific gut microbiota to later life stress exposure. This part of the results may support the double-hit hypothesis of psychopathology, demonstrating that early life stress sensitises at least a part of gut microbiota to later life stress exposure.

Adult stress exposure alone also adversely affected normal balance of gut microflora as indicated by declined counts of aerobic bacteria and beneficial organisms bifidobacteria while increased counts of potential deleterious bacteria such as *Bacteroides* and *E. coli*. This is consistent with previous research showing that exposure of adult mice and rats to restraint stress lead to an imbalance in their gut microflora [63],[64].

Perinatal maternal administration of probiotics appeared to restore the altered gut anaerobic bacteria, bifidobacteria and *E. coli* in stressed adults to one resembling that of no-stress animals. It could be expected that normalisation of gut flora is mediated via increased luminal IgA levels in animals born to probiotic-treated mothers.

Interestingly neonatally stressed rodents born to the probiotic treated dams exhibited increased levels of enterococci and clostridia when exposed to stress in adulthood. This would appear to suggest that early life stress coupled with microbial exposure even with beneficial bacteria could induce long-lasting alterations in the gut microbiota.

Finally, this study does raise concerns about the perinatal use of probiotics given the evidence that they are capable of activating stress pathways. Notably, this was however associated with enhanced immune responses, and to some extent, protection of adult rats against abnormalities in the composition of gut microflora provoked by early and/or later life stress. It is possible that probiotics are perceived as immune challenges inducing long-lasting alterations to the brain-gut axis function. Underlying mechanisms of action however need to be further investigated. Moreover, further efforts to examine the effect of maternal probiotic intervention on other aspects of brain-immune-gut axis integrity and functioning under stress conditions are required.
